# Effects of body position during cardiopulmonary exercise testing with right heart catheterization

**DOI:** 10.14814/phy2.13945

**Published:** 2018-12-11

**Authors:** Saiko Mizumi, Ayumi Goda, Kaori Takeuchi, Hanako Kikuchi, Takumi Inami, Kyoko Soejima, Toru Satoh

**Affiliations:** ^1^ Division of Cardiology Department of Medicine Kyorin University Hospital Mitaka Tokyo Japan

**Keywords:** Cardiopulmonary exercise testing, position, pulmonary circulation, right heart catheterization

## Abstract

Cardiopulmonary exercise testing (CPX) with right heart catheterization (RHC) widely used for early diagnosis and evaluation of pulmonary vascular disease in patients with pulmonary arterial hypertension and early stage heart failure with preserved ejection fraction, who display normal hemodynamics at rest. The aim of this study was to investigate that whether body position affects pulmonary hemodynamics, pulmonary arterial wedge pressure (PAWP), and CPX parameters during invasive CPX. Seventeen patients (58 ± 14 years; 5/12 male/female) with chronic thromboembolic pulmonary hypertension treated with percutaneous transluminal pulmonary angioplasty and near‐normal pulmonary artery pressure (PAP) underwent invasive CPX twice in supine and upright position using a cycle ergometer with 6 months interval. The mean PAP (peak: 45 ± 7 vs. 40 ± 11 mmHg, *P* = 0.006) and PAWP (peak: 17 ± 4 vs. 11 ± 7 mmHg, *P* = 0.008, supine vs. upright, respectively) throughout the test in supine position were significantly higher compared with in upright position, because of preload increase. However, transpulmonary pressure gradient, pulmonary vascular resistance, and mPA‐Q slope during exercise were of no significant difference between two positions. There were no differences between the results of two positions in peak VO_2_ (15.9 ± 4.0 vs. 16.6 ± 3.2 mL/min per kg, *P* = 0.456), the VE versus *V*CO_2_ slope (37.8 ± 9.2 vs. 35.9 ± 8.0, *P* = 0.397), or the peak work‐rate (79 ± 29 vs. 84 ± 27W, *P* = 0.118). Body position had a significant influence on PAP and PAWP during exercise, but no influence on the pulmonary circulation, or peak *V*O_2_, or VE vs.*V*CO_2_ slope.

## Introduction

Cardiopulmonary exercise testing (CPX) with right heart catheterization (RHC) (invasive CPX) attracts a great deal of interest in the area of pulmonary vascular disease in patients with pulmonary arterial hypertension (PAH) and heart failure with preserved ejection fraction (HFpEF) (Borlaug et al. 2010; Andersen and Borlaug 2014). Exercise‐induced pulmonary arterial pressure (PAP), pulmonary arterial wedge pressure (PAWP), pulmonary vascular resistance (PVR) elevations, and measurement of PA pressure‐flow relationships can be evaluated by invasive CPX in these patients.

Early detection of pulmonary vascular dysfunction in PAH patients is an important strategic objective against a terrible disease whose mortality remains high, despite current medical progress. In general, it is not until more than 60% of the pulmonary arteries are obstructed (effective pulmonary flow is less than 40%) that a rise in resting PAP is detected. In normal subjects, pulmonary vasodilatation and reduction in PVR occur during exercise. In advanced or occult pulmonary vascular dysfunction, there is loss of PVR reduction or even an apparent increase in PVR, with a drop in pulmonary arterial compliance. The functional pulmonary arterial bed is destroyed and the arterial bed reserved for recruitment during exercise is already consumed at rest. This is the reason why exercise‐induced raise of PAP suggests the existence of early pulmonary vascular dysfunction in patients with PAH. The future paradigm of early disease detection in high‐risk patients should ideally be aimed at detecting disease before a rise in resting PAP (Lau et al. 2011).

Pulmonary endaterectomy (PEA) and percutaneous transluminal pulmonary angioplasty (PTPA) provides a potential cure for patients with chronic thromboembolic pulmonary hypertension (CTEPH). However, successfully operated patients can continue to suffer from a limitation of exercise capacity, despite normalization of pulmonary artery pressure and PVR. Bonderman et al. (2011) reported that, after successful PEA, patients with persistent exertional dyspnea display an abnormal pulmonary hemodynamic response to exercise, characterized by increased PVR. Exercise testing gives useful information also in CTEPH patients (Claessen et al. 2015; Richter et al. 2015).

The identification of the patients with heart failure with pulmonary vascular dysfunction has recently been increasing interest, too. In some patients HFpEF hemodynamics with/without pulmonary vascular dysfunction is apparent at rest but in others it is only provoked or demonstrated with the stress of exercise. Exercise testing also allows direct measurement of PA pressure‐flow relationships, which are believed to provide greater insight into the extent of pulmonary vascular disease present in a given patient when compared with steady state measurements of hemodynamics. Presence of pulmonary vascular dysfunction will influence on disease prognosis and treatment strategy. The detection of pulmonary vascular dysfunction would become more important in the future.

Exercise provides the most robust and physiologically relevant stressor and can be performed safely in the supine and upright positions in virtually all patients. From previous reports, as an increase in PAWP to greater than or equal to 25 mHg in supine position or ≥20 mmHg in upright position is a sufficient evidence to make the diagnosis of HFpEF (Tolle et al. 2008; Borlaug et al. 2010; Andersen and Borlaug 2014).

Invasive hemodynamic exercise testing had emerged as the gold standard to diagnose or exclude HFpEF in patients with exertional dyspnea of unclear etiology, and is useful for early detection of pulmonary vascular dysfunction in PAH and HFpEF patients, but the method of measuring is still uncertain. Zero level of right heart catheterization in supine position can measure more accurate, on the other hand, sitting position is more physiological for exercise.

The aim of this study was to investigate that whether pulmonary hemodynamics, PAWP and CPX parameters are affected by body position during invasive CPX in the patients with the treated CTEPH, who had near‐normal pulmonary artery pressure at rest.

## Materials and Methods

This study was approved by the committee for clinical studies and ethics of Kyorin University School of Medicine (Approval NO: 490).

### Study patients

Seventeen patients (58 ± 14 years; 5/12 male/female) with chronic thromboembolic pulmonary hypertension (CTEPH) treated with percutaneous transluminal pulmonary angioplasty 6 months earlier with resultant near‐normal PAP (<30 mmHg at rest) were eligible for the study (Kataoka et al. 2012; Inami et al. 2016).

The purposes and risks of the study were explained to the patients, and informed consent was obtained from each patient.

### Right heart catheterization and cardiopulmonary exercise testing

Right heart catheterization was performed with a 6F double‐lumen, balloon‐tipped, flow directed catheter (Harmac Medical Products, Inc., USA) via a transjugular approach.

Baseline hemodynamic data were recorded; the zero reference level (midaxilla) was checked at the beginning of pressure measurement, and PAWP was obtained as the mean value of the occlusion arterial trace. Measurements were obtained at the end of a normal expiration with the patient in the flat position, in order to assess right chamber and pulmonary artery pressure (mean PAP, systolic PAP and diastolic PAP) and PAWP.

Invasive CPX was performed at 6 month intervals without therapeutic intervention. An incremental symptom‐limited exercise test was performed in the supine and upright position, with an electromagnetically braked cycle ergometer (Nuclear Imaging Table with Angio Ergometer; Lode; Groningen, Netherlands) according to the Ramp protocol. Supine testing was performed first and upright testing at 6 months later.

For cycling in supine position, the seat and upper part of the ergometer were set in horizontal and the crank axis was set above the body. The legs were elevated about 30 degrees. The test consisted of a 3‐min resting period, followed by 3 minutes of warm‐up at an ergometer setting of 10 W (60 rpm), followed by testing with a 1 W increase in exercise load every 6 sec (10 W/min).

Oxygen consumption (*V*O_2_), carbon dioxide output (*V*CO_2_), minute ventilation (VE), and end‐tidal CO_2_ (PETCO_2_) were measured throughout the test using a metabolic cart (Cpex‐1; Inter‐Reha Co., Ltd.; Tokyo, Japan). Prior to calculating the parameters from respiratory gas analysis, eight‐point moving average of the breath‐by‐breath data was obtained. The ratios of VE to *V*O_2_ (VE/*V*O_2_) and VE to *V*CO_2_ (VE/*V*CO_2_) and the respiratory exchange ratio (R = *V*CO_2_/*V*O_2_), and PETCO_2_ were computed simultaneously and displayed together with *V*O_2_ on the monitor of a personal computer. The anaerobic threshold (AT) was determined mainly by the V‐slope method and was also identified by the following conventional criteria: (1) VE/VO_2_ increases after being stable or decreasing while VE/VCO_2_ remains constant or decreases, and (2) the respiratory exchange ratio, which has been stable or slowly rising, begins to increase more steeply. Peak *V*O_2_ was defined as the average value obtained during the last 30 sec of incremental exercise. The respiratory compensation point was determined at the point where PETCO_2_ started to decrease. The slope of the increase in ventilation to the increase in *V*CO_2_ (VE vs. *V*CO_2_ slope) was calculated from the start of incremental exercise to the respiratory compensation point by least squares linear regression.

Arterial blood pressure (BP) directly recorded in the radial artery and electrocardiogram and heart rate (HR) were monitored continuously during the test.

The pressure transducer was leveled using as reference the mid axillary line (supine) and 10 cm below the upper edge of manubrium of sternum (upright). PAP and PAWP in RHC were also measured every minute during the test. Transpulmonary pressure gradient (TPG) was defined as subtraction of PAWP from mean PAP (mean PAP – PAWP). Oxygen saturation in arterial blood (SaO_2_), partial pressure of arterial O_2_ (PaO_2_), arterial CO_2_ (PaCO_2_) in the radial artery, and O_2_ saturation in the pulmonary artery (SvO_2_) were measured at rest, AT, and at peak exercise. Cardiac output (CO) was determined by the Fick method using the following formula: CO (L/min) = *V*O_2_/{1.34 × hemoglobin x (SaO_2_‐ SvO_2_)}. Pulmonary vascular resistance (PVR) was calculated as: PVR (Wood units) = (mean PAP – PAWP)/CO. The slope of mean PAP ‐ flow relationship (mPA‐Q slope) was calculated from three point plots of mean PAP and CO by least squares linear regression. All measurements during exercise testing were performed without supplemental oxygen. Six minute walking distance (6MWD) was measured according to American Thoracic Society guidelines. Brain natriuretic peptide (BNP) was assessed in each patient.

### Statistical analysis

The data are presented as the mean ± SD, or median (25th, 75th interquartile range where appropriate. The Shapiro‐Wilk test was used to assess the normality of distribution of the data. All the continuous values, except for BNP, were distributed normally. Number of the studied patients was 17, thus the Wilcoxon signed rank test was used to compare variables between two positions. Statistical comparisons were considered significant at a probability value < 0.05. All analyses were performed using the SPSS statistical package, version 11.0 (SPSS Inc., Chicago).

## Results

### Baseline right heart catheterization

Baseline characteristics of the study patients at resting state in flat position before setting are shown in Table 1. There were no significant differences between two positions, although there was an interval of 6 months. 6MWD and BNP also are comparable between two tests.

**Table 1:** Baseline characteristics at resting state in flat position (before exercise position)

	Supine testing	Upright testing
Mean RA, mmHg	4 ± 3	4 ± 2
Mean PAP, mmHg	20 ± 4	19 ± 4
PAWP, mmHg	10 ± 3	9 ± 3
Cardiac Output, L/min	5.2 ± 1.9	5.1 ± 1.7
PVR, wood unit	2.3 ± 1.1	2.2 ± 1.1
PaO_2_, Torr	74.9 ± 16.4	80.7 ± 18.0
PaCO_2_, Torr	38.0 ± 5.4	38.4 ± 5.3
SaO_2_, %	94.5 ± 3.2	95.3 ± 2.3
SvO_2_, %	73.3 ± 4.8	72.9 ± 4.1
BNP, pg/dL	23.1 (12.0, 44.9)	15.8 (12.0, 35.3)
6MWD, m	498 ± 84	509 ± 88

Values are reported Mean ± SD, or median (25th, 75th interquartile range where appropriate.

Mean RA, Mean right atrium pressure; Mean PAP, Mean pulmonary artery pressure; PAWP, Pulmonary Artery Wedge Pressure; PVR, Pulmonary Vascular Resistance; 6MWD, Six Minute Walking Distance

### Exercise data

The respiratory exchange ratio (R = *V*CO_2_/*V*O_2_) at peak exercise was >1.0 in all subjects suggesting that, at least, sufficient amount of exercise was performed. The R at peak exercise was not significantly different among the two positions (1.09 ± 0.10 vs. 1.11 ± 0.10, *P* = 0.256).

### Pulmonary and systemic hemodynamics during exercise

**Figure 1:**
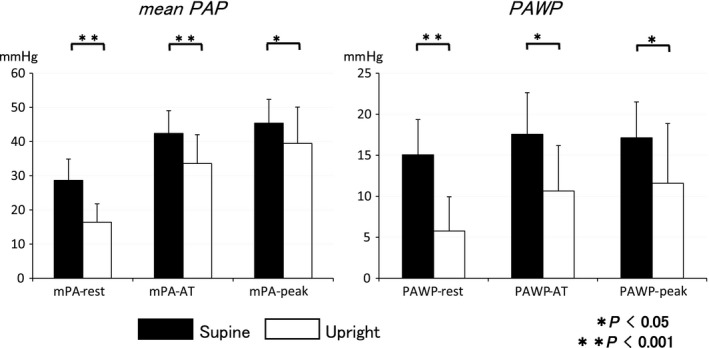
Response of mean pulmonary arterial pressure (PAP) and pulmonary arterial wedge pressure (PAWP) in supine and upright positions at rest, AT and peak exercise. Mean PAP and PAWP in supine position were higher compared with upright position. Body position affected mean PAP and PAWP.

The effects of the different body positions on pulmonary hemodynamic variables are shown in Table 2 and Figure 1. Mean PAP and PAWP increased from 20 to 29 mmHg, and 10 to 15 mmHg in supine testing, respectively, when the individuals went from the flat position to the supine CPX position with the legs elevated approximately 30 degrees. However, in upright testing, there were no differences of mean PAP (from 19 to 16 mmHg) and PAWP (from 9 to 6 mmHg) from flat position to upright CPX position.

**Table 2:** Cardiopulmonary exercise parameters

	Supine testing	Upright testing	*P* value
Rest			
HR, bpm	71 ± 9	68 ± 8	0.220
Systolic BP, mmHg	134 ± 20	141 ± 17	0.130
Diasolic BP, mmHg	69 ± 9	67 ± 14	0.485
Mean BP, mmHg	93 ± 14	94 ± 14	0.569
Systolic PAP, mmHg	50 ± 12	31 ± 7	<0.001
Diastolic PAP, mmHg	15 ± 8	6 ± 5	0.003
Mean PAP, mmHg	29 ± 6	16 ± 5	<0.001
PAWP, mmHg	15 ± 4	6 ± 4	<0.001
TPG (Mean PA‐PAWP), mmHg	14 ± 5	11 ± 5	0.035
Cardiac Output, L/min	6.1 ± 1.7	5.0 ± 1.4	0.020
PVR, wood unit	2.4 ± 1.1	2.4 ± 1.4	0.964
PaO_2_, Torr	73.1 ± 7.3	85.0 ± 16.0	0.021
PaCO_2_, Torr	39.1 ± 5.2	38.8 ± 9.0	0.733
SaO_2,_ %	94.5 ± 2.6	96.7 ± 1.6	0.002
SvO_2,_ %	70.7 ± 5.4	71.0 ± 5.5	0.712
*V*O_2_, mL/min	239 ± 70	208 ± 55	0.040
*V*CO_2_, mL/min	234 ± 137	177 ± 50	0.049
R	0.85 ± 0.10	0.85 ± 0.06	0.865
VE, L/min	11.5 ± 8.3	8.5 ± 2.6	0.051
VE/*V*O_2_	44.0 ± 12.4	42.7 ± 8.9	0.927
VE/*V*CO_2_	52.4 ± 14.0	50.0 ± 8.4	0.579
*Submaximal Exercise (AT)*			
Work Rate, Watt	45 ± 15	44 ± 16	0.964
HR, bpm	107 ± 15	110 ± 11	0.434
Systolic BP, mmHg	155 ± 26	183 ± 23	0.002
Diasolic BP, mmHg	87 ± 21	85 ± 15	0.842
Mean BP, mmHg	114 ± 22	117 ± 19	0.266
Systolic PAP, mmHg	70 ± 14	61 ± 14	0.008
Diastolic PAP, mmHg	21 ± 6	11 ± 7	<0.001
Mean PAP, mmHg	42 ± 7	34 ± 8	<0.001
PAWP, mmHg	18 ± 5	11 ± 6	0.008
TPG (Mean PA‐PAWP), mmHg	24 ± 7	22 ± 9	0.305
Cardiac Output, L/min	10.9 ± 3.2	10.7 ± 2.5	0.782
PVR, wood unit	2.4 ± 1.0	2.2 ± 0.9	0.252
PaO_2_, Torr	73.1 ± 7.3	85.0 ± 16.0	0.002
PaCO_2_, Torr	42.2 ± 8.0	39.8 ± 3.5	0.520
SaO_2_, %	91.0 ± 4.7	92.6 ± 2.6	0.059
SvO_2_, %	49.3 ± 7.1	49.6 ± 5.8	0.940
VO_2_, mL/min	733 ± 236	754 ± 187	0.517
VCO_2_, mL/min	234 ± 137	177 ± 50	0.266
R	0.97 ± 0.07	0.98 ± 0.05	0.687
VE, L/min	29.4 ± 8.6	26.1 ± 7.1	0.207
VE/*V*O_2_	41.0 ± 10.2	35.8 ± 7.0	0.009
VE/*V*CO_2_	43.2 ± 8.2	38.8 ± 7.1	0.001
Peak exercise			
Work Rate, Watt	79 ± 29	84 ± 27	0.055
HR, bpm	128 ± 18	139 ± 16	0.010
Systolic BP, mmHg	170 ± 26	196 ± 29	0.004
Diasolic BP, mmHg	88 ± 20	90 ± 25	0.740
Mean BP, mmHg	116 ± 20	130 ± 21	0.008
Systolic PAP, mmHg	75 ± 15	72 ± 21	0.393
Diastolic PAP, mmHg	21 ± 7	12 ± 9	0.004
Mean PAP, mmHg	45 ± 7	40 ± 11	0.006
PAWP, mmHg	17 ± 4	11 ± 7	0.008
TPG (Mean PA‐PAWP), mmHg	28 ± 9	27 ± 10	0.801
Cardiac Output, L/min	12.5 ± 4.7	12.3 ± 3.4	0.890
PVR, wood unit	2.7 ± 1.2	2.4 ± 0.9	0.243
PaO_2_, Torr	61.7 ± 10.0	67.1 ± 9.7	0.015
PaCO_2_, Torr	38.3 ± 5.6	37.8 ± 4.4	0.147
SaO_2_, %	89.2 ± 5.4	90.9 ± 3.5	0.102
SvO_2_, %	42.8 ± 7.0	42.0 ± 6.2	0.737
*V*O_2_, mL/min	953 ± 344	997 ± 316	0.548
*V*CO_2_, mL/min	1041 ± 402	1105 ± 389	0.378
R	1.09 ± 0.10	1.11 ± 0.10	0.256
VE, L/min	42.0 ± 13.2	42.1 ± 13.9	0.988
VE/*V*O_2_	46.0 ± 8.2	43.2 ± 9.4	0.263
VE/*V*CO_2_	42.8 ± 7.1	38.9 ± 7.3	0.004
Peak VO_2,_ mL/kg per min	15.9 ± 4.0	16.6 ± 3.2	0.548
VE versus *V*CO_2_ slope	37.8 ± 9.2	35.9 ± 8.0	0.263
mPA‐Q slope	3.6 ± 2.2	3.2 ± 1.3	0.378

Values are means Mean ± SD.

HR, Heat Rate; BP, Blood Pressure; PAP, Pulmonary Artery Pressure; PAWP, Pulmonary Artery Wedge Pressure; TPG, Transpulmonary Pressure Gradient; PVR, Pulmonary Vascular Resistance; VO_2_, Oxygen Consumption; VCO_2_, carbon dioxide output; R, respiratory exchange ratio; VE, minute ventilation.

Through the exercise test, mean PAP and PAWP in supine position were higher compared with upright position. However, TPG, PVR, and mPA‐Q slope during exercise were of no significant difference between the two positions. Partial pressure of arterial O_2_ during exercise was significantly lower in supine position.

Resting arterial BP and HR were of no significant difference between the two positions, but arterial pressure at AT and peak and HR at peak were significantly higher in upright position. Cardiac output at rest was significantly higher in the supine position than that in the upright position, however, CO at AT and peak were of no difference.

### Cardiopulmonary exercise testing

**Figure 2:**
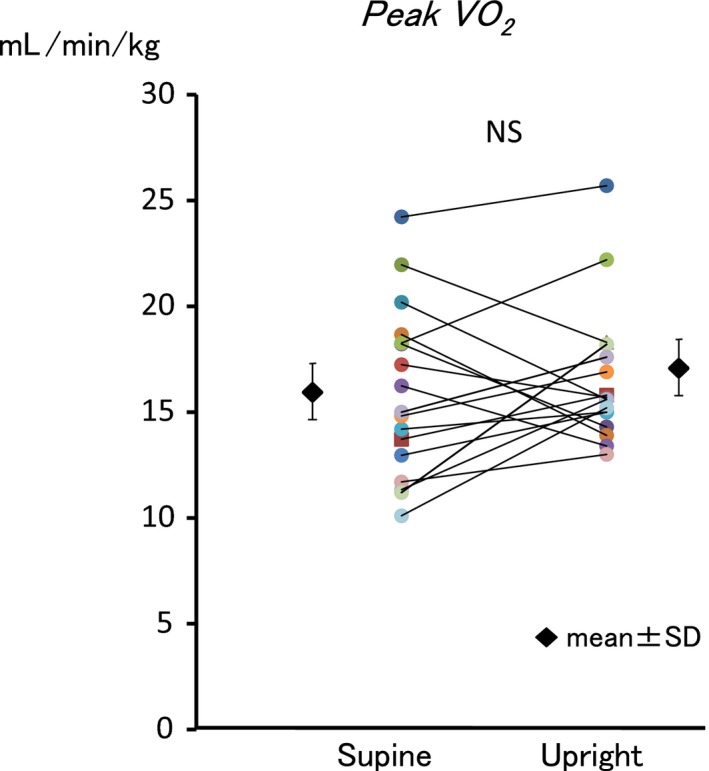
Comparison between peak *V*O_2_ in supine and upright positions. Body position did not affect peak *V*O_2_.

The effects of different body positions on CPX variables at each exercise stage were shown in Table 2. There were of no differences between the results in the supine position and the upright position in peak work‐rate (79 ± 29 vs. 84 ± 27 watt, *P* = 0.055). Peak *V*O_2_ (15.9 ± 4.0 vs. 16.6 ± 3.2 mL/min per kg, *P* = 0.548) and *V*O_2_ at AT were not affected by the positions (Table 2 and Fig. 2).

**Figure 3:**
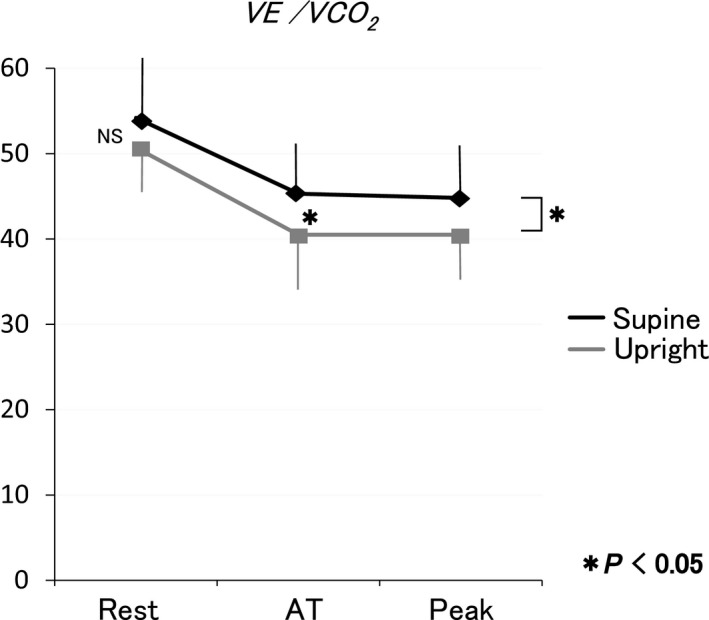
Response of VE/*V*CO_2_ during exercise in supine and upright positions. VE/*V*CO_2_ in supine position was greater than that in upright position. VE/*V*O_2_ curve shifted parallel.

**Figure 4:**
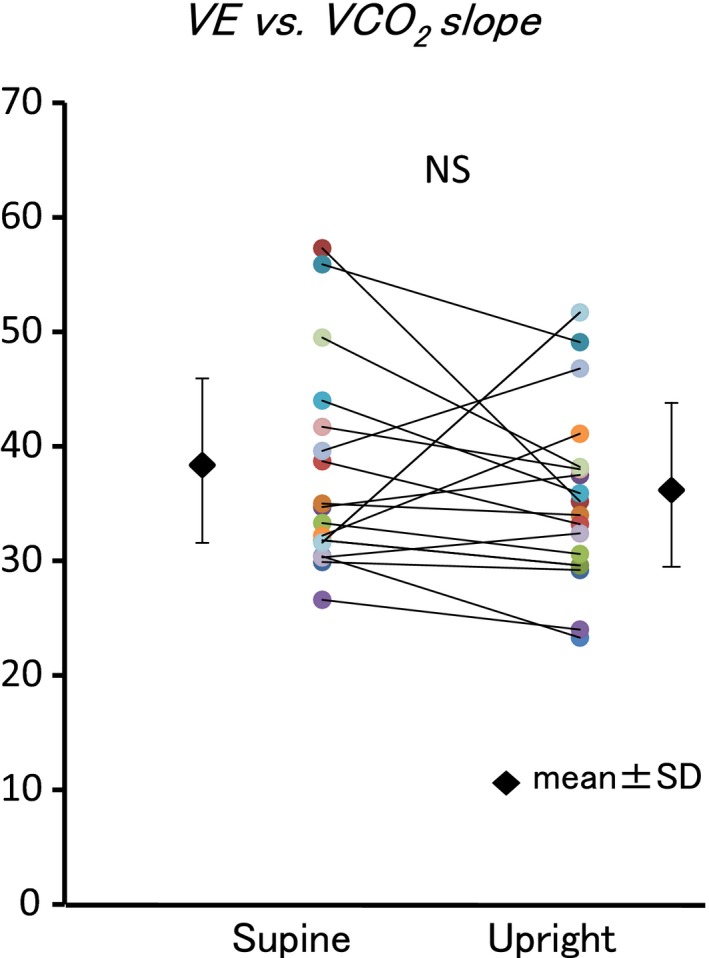
Comparison between VE versus *V*CO_2_ slope in supine and upright positions. Body position did not affect VE versus *V*CO_2_ slope.

VE/VCO_2_ was greater in supine position during the exercise (Table 2). VE/*V*O_2_ curve shifted parallel (Fig. 3). Therefore, VE versus *V*CO_2_ slope (37.8 ± 9.2 vs. 35.9 ± 8.0, *P* = 0.263) did not change (Fig. 4).

## Discussion

The present study reveals that body position does not affect pulmonary hemodynamics, peak *V*O_2_, AT *V*O_2_, and VE versus *V*CO_2_ slope though PAWP and resultant PAP increase throughout exercise in the supine position due to preload increase.

### Pulmonary circulation and pulmonary arterial wedge pressure

In our study, body position affects PAP, because of elevation in PAWP. Leg raise in the supine position caused an increase in venous return to heart. Elevation of PAWP caused PAP elevation through postcapillary mechanics. However, TPG, PVR, and mPA‐Q slope were not influenced by body position, that is, pulmonary circulation is unaffected by posture. Similar body position‐dependent changes also have been previously noted. Forton et al. (2016) also reported that pulmonary circulation was unaffected by posture.

Reeves et al. and Kovacs et al. (2012) reviewed the behavior of PVR and its different patterns during supine and upright exercise. During supine position, they described a minimal PVR decrease. On the other hand, the upright position at rest is associated with a lower cardiac output, derecruitment of the pulmonary circulation, unchanged mean PAP because of pulmonary vascular closure, and thus increased PVR (Wong et al. 2014). However, during the exercise, pulmonary resistive vessels in fully recruited lungs would be reopened leading to a fall in PVR (Harf et al. 1978). PVR continues to decrease with increasing levels of exercise independently of body position. This is explained by the natural distensibility of the pulmonary circulation, that is, the normal pulmonary vascular bed is a low‐pressure, low‐resistance, highly distensible system that can adapt to a large increase in blood flow, such as during physical exercise, with minimal elevation of PAP. Another explanation of different PVR behavior is that vasoconstrictive mechanisms are activated at rest in the upright position that allow for a relatively even perfusion of all parts of the lung and lead to an elevated PVR. This vasoconstriction would be released during exercise resulting in a PVR decrease. Our resting PVR in upright position was comparable with supine positions. This might be caused natural distensible system.

From previous reports, as increase in PAWP to greater than or equal to 25 mmHg in supine position or ≥20 mmHg in upright position is a sufficient evidence to make the diagnosis of HFpEF (Andersen and Borlaug 2014; Esfandiari et al. 2017; Naeije et al. 2018). The difference of PAWP between two positions is 5 mmHg. In our study, PAWP at peak exercise in the sitting position was 11.3 mmHg and in the supine position was 17.1 mmHg, and the difference between the two positions was about 5.8 mmHg.

### Ventilatory response and exercise capacity

With CPX alone, it is usually done in the upright position, the physiological status. The lower lung zones have two and half times more the ventilation and five times more the blood flow of the upper zone in the upright position than in the sitting position, suggesting that gravity has a greater effect on blood flow than on regional ventilation (Bryan et al. 1964). In the upright lung, ventilation and perfusion are nonuniform with increased ventilation relative to perfusion at the apices. In supine lung, ventilation perfusion matching throughout the lung is practically more uniform.

VE versus *V*CO_2_ slope, which reflects a ventilatory efficiency during exercise, has been emphasized as a powerful predictor of prognosis and severity of heart failure and PAH (Chua et al. 1997; Schwaiblmair et al. 2012). VE versus *V*CO_2_ slope is considered to be derived from the ventilation‐perfusion mismatch and the increased ratio of physiologic dead space to tidal volume, which are mainly due to the inappropriate increase in cardiac output during exercise. An increase in dead space ventilation has been proposed as a principle reason for the increase in VE/*V*CO_2_.

From the past reports, postural effect of ventilatory efficiency is unclear. No effect on VE versus VCO_2_ slope by posture was seen in one report (Takahashi et al. 1998), on the other hands, VE versus *V*CO_2_ slope in supine position was lower than that in upright position in other reports (Armour et al. 1998; Terkelsen et al. 1999). The present study shows that the VE versus *V*CO_2_ slope is unaffected by posture.

In our study, VE/*V*CO_2_ in the supine position tend to be higher than that in upright position due to increased dead space ventilation. VE/*V*CO_2_ curve shifted parallel between supine and upright position. As a result, VE versus *V*CO_2_ slope did not change. Because of the increase in physiological dead space ventilation, minute ventilation is increased in patients with pulmonary vascular disease at rest, and to greater degree during exercise. This might be a reason that VE/*V*CO_2_ was higher in the supine position. In some of our patients, PAP and PAWP were elevated by exercise. It caused different results from normal subjects. In our study, peak *V*O_2_ and peak work‐rate tended to be lower in the supine position, but no significant differences were observed. In the previous reports, *V*O_2_ throughout the test and peak *V*O_2_ in the supine position was significantly lower than that in upright position (Leyk et al. 1994; MacDonald et al. 1998; Terkelsen et al. 1999; Forton et al. 2016). The higher *V*O_2_ in an upright position is presumably due to the increased exercising muscle mass required to keep the body upright. The reason why our result was different from the previous reports was thought to be more healthy subjects with low peak VO_2_ in our subjects.

### Effects on arterial blood pressure and heart rate

Arterial systolic BP and HR at peak exercise in the upright position were significantly higher than those in the supine position. These results are in keeping with a lot of previous reports of higher systemic blood pressure and heart rate in the upright compared with the supine position. Takahashi et al. (1995) reported that higher plasma noradrenaline and angiotensin II were observed during upright position. It is considered that the BP and HR increase due to systemic nerve and hormonal effects by posture.

In general, cardiac output in upright position is lower than that in supine position (Takahashi et al. 1995; Fraser et al. 2015). Effect of posture on cardiac output during exercise was reported in some studies (Bevegard et al. 1960; Poliner et al. 1980; Higginbotham et al. 1986; Leyk et al. 1994; Trinity et al 2011). Cardiac output at rest in the supine position was significantly higher than that in the upright position in our study. This is due to an increase in stroke volume in the supine position caused by increase in venous return. However, cardiac output at AT and peak exercise was comparable between two positions.

### Limitation

In the interpretation of our study results, some limitations should be considered.

The major limitations were the small number of patients and the lack of a control group. This study was performed in patients with CTEPH. Therefore, these findings may not be broadly generalizable. Since the subjects are not normal, it may not be generalized to normal population. Our two tests have an interval of 6 months, which may affect the results. However, in the two tests, there was no significant difference in hemodynamic parameters and 6MWD, we thought that there was no great difference in their exercise capacity.

## Conclusions

In conclusion, body position had a significant influence on PAP and PAWP during exercise, and no influence on the pulmonary circulation, peak *V*O_2_, or VE versus *V*CO_2_ slope.

## Conflict of Interest

No conflicts of interest, financial or otherwise, are declared by the author(S).
